# Ergonomics in Endoscopy—Editorial

**DOI:** 10.1002/ueg2.70019

**Published:** 2025-04-05

**Authors:** Keith Siau, Swati Pawa

**Affiliations:** ^1^ Royal Cornwall Hospitals NHS Trust Truro UK; ^2^ Wake Forest University School of Medicine Winston‐Salem North Carolina USA

The term ergonomics (Greek: Ergos—works; nomos—laws) translates to ‘the laws of work’ and refers to the interplay between humans and their work environment. Ergonomics is critical in endoscopy as the work requires repetitive motions, prolonged awkward postures, and the application of significant forces, all of which may result in endoscopy‐related injuries (ERIs).

In their systematic review and meta‐analysis (30 studies, 7646 endoscopists), Oliviera et al. estimate a pooled career‐long prevalence of ERI at 62.5%. The hand (28.2%), lower back (27.3%), thumb (27.1%) and neck (25.7%) were predominantly affected. ERIs are therefore common and not only threaten the endoscopist's quality of life, performance and career longevity, but may also affect patient outcomes and service capacity.

Notably, 19 (63%) of the 30 studies were published after 2020, attesting to the recent attention to the problem. These survey‐based studies often relied on non‐validated questionnaires, faced response bias, and lacked standardised definitions of ERIs and control group comparisons. These limitations likely account for the substantial heterogeneity observed in this meta‐analysis. Over half of studies originate from the United States where the American Society for Gastrointestinal Endoscopy (ASGE) has been at the forefront of addressing ergonomics in endoscopy.

In the survey, 43.8% of endoscopists required practice modifications, primarily through adjustments to equipment layout and periprocedural stretching. Simple measures, such as optimizing monitor and table heights, help reduce strain on the neck and lower back. Intraprocedural stretching, microbreaks, anti‐fatigue mats, and ergonomic footwear can further alleviate pain and fatigue. These should be covered within a dedicated ergonomics curriculum for endoscopy trainees and trainers [1]. Ergonomics guidelines such as ASGE guidelines should be developed and implemented to suit the needs of the workforce [2].

Endoscopy demands precision and endurance while wielding a ‘one size fits all’ instrument that has been largely unchanged in design since the 1980s. The pattern of left‐sided hand and thumb injuries particularly among female endoscopists implicates endoscope design and handling and represents a gender disparity. In a recent survey (*N* = 455) [3], 85.7% favoured a pro‐ergonomic colonoscope redesign with customizability for hand size of control knobs (85%) and decreased force required for ‘up/down’ wheel manipulation (78%). These changes were more desirable among female endoscopists. There are therefore calls for the industry to work with endoscopists to develop tools which prioritize ergonomics.

Higher procedural volumes, longer careers, and female gender were risk factors for ERIs. In the era of rising endoscopy demand, increasing retirement ages, and endoscopy becoming a more women‐inclusive specialty, attention to ergonomics is more important than ever. By integrating ergonomic training, optimizing workplace design, and welcoming industry in the discussions, the field can finally address the ‘laws of work’ to prioritize endoscopist safety.

## Conflicts of Interest

The authors declare no conflicts of interest.

## Data Availability

The data that support the findings of this study are available on request from the corresponding author. The data are not publicly available due to privacy or ethical restrictions.
